# Novel synthetic clot analogs for in-vitro stroke modelling

**DOI:** 10.1371/journal.pone.0274211

**Published:** 2022-09-09

**Authors:** Helena Guerreiro, Nadine Wortmann, Thomas Andersek, Tuan N. Ngo, Andreas M. Frölich, Dieter Krause, Jens Fiehler, Anna A. Kyselyova, Fabian Flottmann

**Affiliations:** 1 Department of Diagnostic and Interventional Neuroradiology, University Medical Center Hamburg-Eppendorf, Hamburg, Germany; 2 Institute of Product Development and Mechanical Engineering Design, Hamburg University of Technology, Hamburg, Germany; National University of Ireland - Galway, IRELAND

## Abstract

**Purpose:**

The increased demand for training of mechanical thrombectomy in ischemic stroke and development of new recanalization devices urges the creation of new simulation models both for training and device assessment. Clots properties have shown to play a role in procedural planning and thrombectomy device effectiveness. In this study, we analyzed the characteristics and applicability of completely synthetic, animal-free clots in the setting of an in-vitro model of mechanical thrombectomy for training and device assessment.

**Methods:**

Synthetic clots based on agarose (n = 12) and silicone (n = 11) were evaluated in an in-vitro neurointervention simulation of mechanical thrombectomy with clot extraction devices. Calcified clots of mixed nature were simulated with addition of 3D printed structures. 9 clots were excluded due to insufficient vessel occlusion and failure to integrate with clot extraction device. Synthetic thrombi were characterized and compared using a categorical score-system on vessel occlusion, elasticity, fragmentation, adherence and device integration.

**Results:**

Both agarose-based and silicone-based clots demonstrated relevant flow arrest and a good integration with the clot extraction device. Silicone-based clots scored higher on adherence to the vessel wall and elasticity.

**Conclusion:**

Selected synthetic clots can successfully be implemented in an in-vitro training environment of mechanical thrombectomy. The clots’ different properties might serve to mimic fibrin-rich and red blood cell-rich human thrombi.

## Introduction

Clinical evidence has shown in the past years the undeniable value of mechanical thrombectomy (MT) in the treatment of acute large vessel occlusion (LVO) [1]. This led to a global increase in the demand for interventionalists with technical proficiency in endovascular stroke treatment [2]. Training of neurointerventionalists may be challenging due to the delicate nature of neurovascular interventions and the scarce availability of dedicated stoke simulation models representing realistic stroke-specific interventional challenges. Animal models used for medical training or device testing are associated not only with an ethical dilemma but also with high costs and a poor reproducibility of human anatomy [3]. In this study we use a fully animal-free experimental setting, comprised of a previously described neurointervention simulation model—HAmburg Anatomical NEurointerventional Simulator (HANNES) [4] and custom-designed 3D-printed models of intracranial vasculature as a realistic and cost-effective training environment for mechanical thrombectomy (MT).

Clot composition has been shown to play a role in the etiology and characterization of ischemic stroke in large vessel occlusions [5]. This may play a role in the development and research of new therapy concepts and thrombectomy devices. One previous study states the importance of artificially made thrombi from human or pig´s blood in the pre-evaluation of thrombus extraction devices and as training material [6]. In this study we analyzed the feasibility of completely animal-free, synthetically made clots in the setting of neurovascular simulation of MT and their interaction with extraction devices.

Our hypothesis was that synthetic clots allow for sufficient vessel occlusion and device interaction in an experimental simulation model of mechanical thrombectomy when evaluated by experienced neurointerventionalists, and that different clot compositions may show different mechanical properties which may mimic characteristics found in human clots.

## Material & methods

### Synthetic clot development and selection

Synthetically produced clots (n = 23) mainly composed of agarose (n = 12, A1-12) or silicone (n = 11, S1-11) were tested in a previously described neurovascular simulation model [4]. Different concentrations of these compounds were mixed with a 3:1 mixture of methylchloroisothiazolinone (MCI) and methylisothiazolinone (MI) (C. Kreul GmbH & Co. KG, Hallerndorf, Germany) or/and micro-glass beads (MGB) (Carl Roth GmbH + Co KG, Karlsruhe, Germany) in different proportions in order to simulate different degrees of stiffness, elasticity, adhesion and fragmentation (Table 1). 3D-printed supporting structures, spiral- or barbed-shaped, were found to be most suitable to be integrated in agarose clots (n = 3) in order to simulate the irregularly shaped calcifications found in-vivo (Flexible Resin, Formlabs 2, MA, USA). Failure to cause a sufficient vessel occlusion (e.g., due to distinctive fragility) or interact with the clot extraction device (and thus, affecting sufficient assessment of stent-retrieval suitability) was considered an exclusion criterion. From the 23 clots that were intended for in-vitro testing, 9 clots were excluded: 4 out of 12 agarose-based clots due to insufficient vessel occlusion and failure to integrate with the stent retriever and 1 out of 12 due to failure to integrate with the stent retriever. 4 out of 11 silicone-based clots were excluded due to failure to interact with the stent retriever. Tubular shaped synthetic clots were prepared with a length of 9–11 mm and a diameter of 2.5 mm. Process of clot production is thoroughly described by Wortmann N. et al. [7].

**Table 1:** Summary of selected synthetic clots, their general composition and possible applicability.

Clot	Composition	Potential Application
A2	Agarose 2%, MCI/MI 10%	white clot
A3	Agarose 2%, MCI/MI 10%, spiral supporting structures	mixed calcified clot
A4	Agarose 2%, MCI/MI 10%, barbed supporting structures	white calcified clot
A5	Agarose 2%, MCI/MI 20%	white clot
A7	Agarose 2%, MCI/MI 5%	white clot
A9	Agarose 5%, MCI/MI 10%, supportive structures	white calcified clot
A12	Agarose 5%, MCI/MI 5%	white clot, fragile
S1	Silicone 30%, MCI/MI 30%	red clot
S3	Silicone 30%, MCI/MI 30%, preserved in oil	red clot
S4	Silicone 30%, MCI/MI 40%, MGB 10%	red clot
S5	Silicone 30%, MCI/MI 40%, MGB 20%	mixed clot
S6	Silicone 30%, MCI/MI 40%, MGB 30%	red clot
S9	Silicone 40%, MCI/MI 20%	white clot
S10	Silicone 40%, MCI/MI 30%	white clot

### Neurovascular simulation environment

Synthetic clot testing was performed in a neurovascular simulation environment (HANNES) [4, 8] integrated on a monoplane angiography system (AlluraClarity FD 20, Philips Healthcare, Best, The Netherlands). Commercially available standardized models of femoral vessels and thoracoabdominal aorta (Neuro Testing Model NTM00V02, United Biologics, Santa Ana, CA) were integrated with 3D-printed, patient specific common carotid and internal carotid arteries attached to a skull base prototype as previously described [4, 8]. Intracranial circulation was simulated using a realistic 3D-printed whole brain vascular model based on patient anatomy including anterior and posterior circulation with a right-sided posterior communicating artery. Physiological flow rate, pulse and body-temperature were simulated by an integrated fluid pump, equipped with a pulsatile valve and a heating system. The standard system configuration produced a flow rate of 0.4L/min, a pulse rate of 70 bpm and a system temperature of 37°C. Blood was substituted with a solution of water and small amount of commercially available soap for friction reduction.

### Mechanical recanalization experimental setup

**Fig 1:**
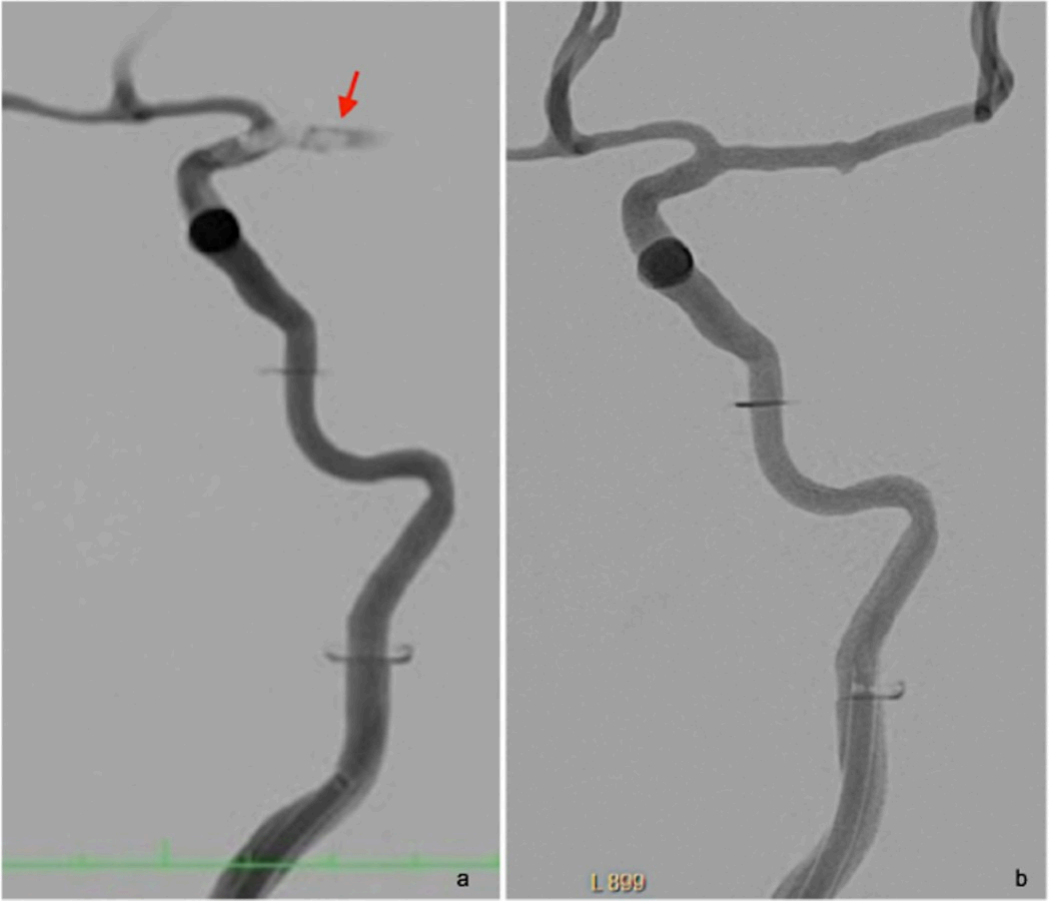
Subtraction angiography of a custom made whole-brain vascular model showing, upon injection of iodinated contrast, a carotid-T occlusion created by an agarose-based synthetic clot (arrow) before (a) and after (b) mechanical recanalization. Note the guide catheter placed in the cervical ICA.

A standard short 8F sheath was placed in the right femoral artery of the experimental model. An 8F-FlowGate guiding catheter (Stryker, MI, USA) was introduced into the internal carotid artery. Clots were directly administered into the common carotid artery of the experimental model via an additional, for this purpose constructed cervical arterial branch. The resulting clot position was evaluated with 10 ml of iodinated contrast medium (Imeron 150, Bracco, Milan, IT) in a single angiographic run (Fig 1). Each clot of the same type was extracted using three different techniques (one recanalization approach per clot): first with aspiration only, using a Sofia 5F or 6F aspiration catheter (Microvention, CA, USA), secondly with a Trevo 6/25 stent retriever (Stryker, MI, USA) and lastly the retrieval maneuver was repeated with a combined approach using the stent retriever and aspiration simultaneously [9]. Finally, selected clots were further evaluated for defined characteristics using a simple silicone tube (5 mm inner diameter; Roth, Karlsruhe, Germany) in order to observe clot interactions without other system factors, such as catheter manipulation, vessel model curvature or inner surface. The clots were positioned within the tube and the interaction with the stent retriever was closely observed and documented.

### Data acquisition and analysis

Clots were qualitatively evaluated in the HANNES model by three neuroradiologists with an average of 5 years-experience in angiography. Evaluation was performed using an ordinal classification scale with a 5-point system (0 to 4) based on qualitative thrombi characteristics with five items: vessel occlusion, elasticity, fragmentation, adherence and device integration (Table 2). This was based on both clinical practice with human thrombi in MT as well as comparatively with other synthetic clots in the study (e.g. elasticity). Data documentation and scoring was performed on Microsoft Excel for Mac (Redmond, WA, USA). Standard descriptive statistics were performed using Prism 9 for macOS Version 9.1.0 (GraphPad Software, LA Jolla, CA, USA). Non-parametric qualitative scale ranks were analyzed using a Mann-Whitney Test performed on MedCalc (MedCalc Software, Ostend, Belgium). Results were displayed in terms of medians and p values. A p-value < 0.05 was considered significant.

**Table 2:** Classification scale for the qualitative evaluation of synthetic clot characteristics in an in-vitro experimental setting.

Criteria	Scoring
Vessel occlusion	0 = none
	1 = negligible flow arrest
Degree of flow arrest on the vessel occluded with a synthetic clot	2 = mild flow arrest
	3 = relevant flow arrest
	4 = complete flow arrest
Elasticity	0 = not elastic
	1 = minimally elastic
	2 = mildly elastic
Elastic deformation of synthetic clots upon interaction with wires and retrieval devices	3 = elastic
	4 = very elastic
Fragmentation	0 = not fragmented
	1 = minimally fragmented
	2 = moderately fragmented
Clot ability to fragment. Associated with higher rate of intraprocedural peripheral embolism	3 = easily fragmented
	4 = very fragmented
Adherence	0 = none
	1 = minimally adherent
	2 = moderately adherent
Measure of the clot’s grip on the vessel wall. It is associated with a decreased probability of clot migration	3 = adherent
	4 = very adherent
Device integration	0 = no integration
	1 = minimal integration
Describes interaction of the clot with the retrieval device. A better device integration is associated with higher therapeutical success rate.	2 = moderate integration
	3 = good integration
	4 = very good integration

## Results

### Thrombus design

Agarose-based thrombi were primarily soft and fragile, tendentially breaking before installation in the vascular model. Addition of MCI/MI showed to significantly reduce their breakability and helped producing compacter structures. Addition of other solid elements such as resins developed and printed in different shapes using 3D-printing techniques showed to be a feasible solution in the production of synthetic models mimicking the behavior of calcified clots of mixed nature. Fragmentation showed to be the main feature of agarose-based clots.

Pure silicone clots presented as too stiff and failed to show the adherence expected form fibrin clots. Realistic slightly more malleable and more adherent clots could be achieved by adding different concentrations of MCI/MI. Added micro-glass beads (MGB) lead to a further increase in fragmentation and adhesion to vascular model walls. Material “stickiness” was effectively reduced through preservation of clot material in an oily substance. Silicone-based clots showed variable degrees of adherence and elasticity as well as reduced fragmentation.

### Thrombus evaluation in an in-vitro setup

**Fig 2:**
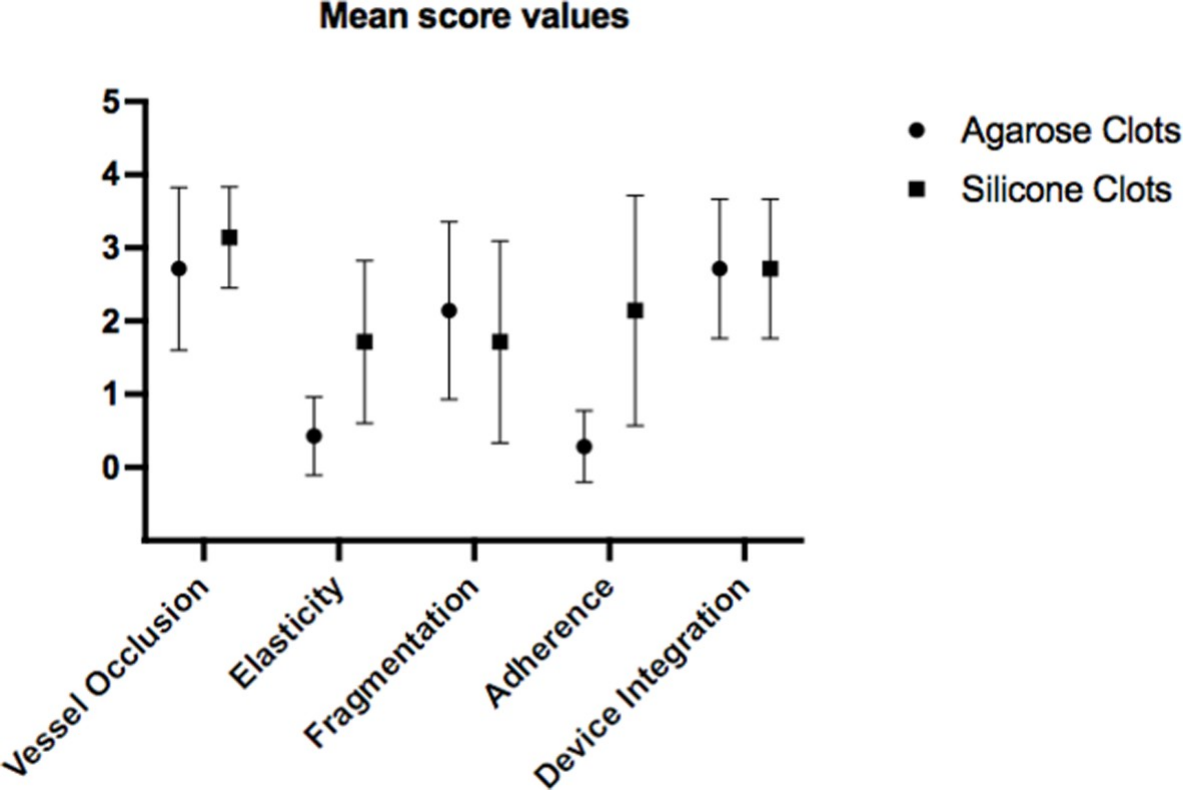
Error bar graph comparing mean score values for agarose- and silicone-based artificial clots. Note specially the different mechanical behaviors concerning clot elasticity and adherence. Both clot groups presented a good to very good vessel occlusion and device integration.

7 agarose-based clots and 7 silicone-based clots were included in the analysis. Both types of clots caused relevant flow arrest and demonstrated a good integration with the clot extraction device (Fig 2). A moderate tendency to fragmentation was observed in both types of clots. Silicone-based clots showed significant higher elasticity (median 2.5 vs 0.5, p = 0.0058) and adherence to the vessel wall (median 3.5 vs 0, p = 0.0048) upon removal with the retrieval device. The majority of clots could be retrieved by aspiration alone (n = 9, 64.3%), 4 out of 7 silicone-based and 5 out of 7 agarose-based clots.

## Discussion

In this experimental study, we developed and tested a novel concept of animal-free synthetic clots for training and research on mechanical thrombectomy, in an in-vitro setting, using a previously well-established neurovascular simulation model [4]. Clots which were unable to provide minimal vessel occlusion or sufficient interaction with retrieval devices were classified as unsuitable for training proposes and were therefore excluded from the analysis. We found that selected agarose-based and silicone-based clots were able to achieve sufficient vessel occlusion and integration with the clot extraction device. Silicone-based clots were more adherent to the vessel wall and more elastic than agarose-based clots.

Berndt et al. proposed a collection of embolus analogs based on human and pig’s blood for device evaluation and training [6]. Other studies described embolus analogs based on a variety of animal and human blood [10] or through addition of synthetic components to human blood [11, 12]. To our knowledge, this study describes the first fully synthetic clot replacement model, free from animal or human blood. An animal-free experimentation setting is increasingly important due to not only the ethical concerns but also in order to establish a simplified and cost-effective solution for regular MT training and device research.

Previous studies have stated the importance of clot histological composition on its mechanical properties and secondarily, on the effectiveness of revascularization techniques in ischemic stroke [13]. Clots can be classified as red/soft clots, if the concentration of red blood cells (RBC) is at least 15% higher than platelets or fibrin, as white/hard clots, if concentration of fibrin is at least 15% higher than RBC and as mixed, if the RBC/fibrin concentration is present in any other proportion [14]. Several studies reported an association of clot composition with the pathophysiology of vascular occlusion: whereas cardioembolic thrombi present with higher mean proportions of fibrin, less RBCs and more WBCs, clots of non-cardioembolic etiologies, such as large artery atherosclerosis or cryptogenic stroke, are mainly associated with RBC-rich clots [15, 16]. However, clot composition remains highly variable, with other factors such as clot age and presence of calcifications, playing a role in their behavior [5].

Development of artificial thrombi with different properties is important for a realistic simulation of MT. In this study we describe artificial clots that potentially mimic the natural behaviors found in red, white, mixed or calcified clots. Red or RBC-rich clots present predominantly with a viscoelastic soft texture [17] and higher friability [18]. Clot permeability or perviousness is defined by the degree to which blood is able to flow through a vessel occlusion. RBC-rich thrombi are associated with higher perviousness and clot attenuation in CT [19, 20], which showed to correlate with better technical and clinical outcomes of patients undergoing MT [21]. Further studies have demonstrated that red clots are associated with reduced number of recanalization maneuvers and overall reduced procedural time [15].

White or fibrin-rich clots have been described as stiffer, presenting increased friction or adherence on the vessel wall [22]. Aged thromboemboli are especially compact structures, lacking elasticity and increasingly prone to fragmentation [10]. White clots present with lower attenuation on non-contrast-enhanced CT and increased resistance to both intravenous lysis and mechanical thrombectomy [23]. Technical aspects of MT such as speed of retrieval [24] or device selection [15] may be improved based on clot composition [20, 23, 25]. Namely utilization of stent retriever in MT seems to be less effective in the case of calcified thromboemboli [26].

The agarose-based clots presented in this study may mimic white, mostly aged clots, showing reduced elasticity and fairly high breakability. However, the expected adherence of such clot materials fails to be met. On the other hand, highly elastic silicone-based clots with variable degrees of breakability presented with high level of adherence. Nonetheless, in this study clot adherence did not affect the technical success of MT. Further developments on clot analogs composition are necessary to optimize artificially composed thrombi and to make them easily available to a higher number of neurovascular centers involved in the research and training of MT.

The exploratory study design with qualitative data interpretation is typically subject to researcher confirmation bias, which may influence the results and make the data less replicable. The neurointerventionalists were not blinded to clot type upon their evaluation. Inter-observer differences could be overcome with further development of objective quantitative assessment methods in this in-vitro set up. Furthermore, an exact comparison between synthetic and human clots can only be achieved by comparing their mechanical properties.

## Conclusion

Selected synthetically composed clot analogs achieved sufficient vessel occlusion and integration with the clot extraction device in an in-vitro stroke thrombectomy model. The characteristic properties of agarose-based and silicone-based clots may be used to mimic different types of human thrombi. Synthetic clots could effectively be used for device testing, as well as for training of endovascular treatment of ischemic stroke.

## Supporting information

S1 Table Qualitative scores obtained for each of the 23 evaluated clots. Clots that failed to interact with the clot removal device (Score = 0 for the category *device integration)* were classified as not suitable for an in-vitro experimental setting and thus excluded (marked in grey, n = 9).
(DOCX)

S1 Fig (a-d). Silicone-based clots mixed with 30% MCI/MI with (c,d) and without micro-glass beads (a,b): before (a,c) and after (b,d) mechanical sheer stress showing different breakability and elasticity degrees.
(DOCX)

S2 Fig (a-c). Retrieved clots with stent retriever. Agarose-based clots mixed with 10% MCI/MI (a, c), with spiral (a) or barbed (c) supporting structures, and with 20% MCI/MI (b) were successfully retrieved with a clot extraction device (SolitaireTM 4mm x 20mm, Medtronic, Dublin, Ireland).
(DOCX)

## References

[1] Goyal M, et al; Endovascular thrombectomy after large-vessel ischaemic stroke: a meta-analysis of individual patient data from five randomised trials. The Lancet 2016; 10029 (387): 1723–1731. doi: 10.1016/S0140-6736(16)00163-X 26898852

[2] Zaidat OO, Lazzaro M, McGinley E, et al. Demand-supply of neurointerventionalists for endovascular ischemic stroke therapy. Neurology. 2012;79(13 Suppl 1): S35–41. doi: 10.1212/WNL.0b013e31826957ef 23008409

[3] Gralla J, Schroth G, Remonda L, et al. A Dedicated Animal Model for Mechanical Thrombectomy in Acute Stroke. American Journal of Neuroradiology. 2006;27(6):1357–1361. 16775297 PMC8133935

[4] Spallek J, Kuhl J, Wortmann N, et al. Design for Mass Adaptation of the Neurointerventional Training Model HANNES with Patient-Specific Aneurysm Models. Proceedings of the Design Society: International Conference on Engineering Design. 2019;1(1):897–906. doi: 10.1017/dsi.2019.94

[5] Bacigaluppi M, Semerano A, Gullotta GS, Strambo D. Insights from thrombi retrieved in stroke due to large vessel occlusion. J Cereb Blood Flow and Metabolism. 2019;39(8):1433–1451. doi: 10.1177/0271678X19856131 31213164 PMC6681524

[6] Berndt M, Prothmann S, Maegerlein C, et al. Artificial Stroke Clots: How Wide is the Gap to the Real World? World Neurosurgery. 2018;110:e90–e99. doi: 10.1016/j.wneu.2017.10.090 29107162

[7] Wortmann N; Andersek T; Guerreiro H; Kyselyova AA; Frölich AM; Fiehler J; et al. Development of synthetic thrombus models to simulate stroke treatment in a physical neurointerventional training model; All Life; 15:1, 283–301. doi: 10.1080/26895293.2022.2046181

[8] Nawka MT, Spallek J, Kuhl J, et al. Evaluation of a modular in vitro neurovascular procedure simulation for intracranial aneurysm embolization. J NeuroIntervent Surg. 2020;12(2):214–219. doi: 10.1136/neurintsurg-2019-015073 31320551

[9] Maus V, Behme D, Kabbasch C, et al. Maximizing First-Pass Complete Reperfusion with SAVE. Clin Neuroradiol. 2018;28(3):327–338. doi: 10.1007/s00062-017-0566-z 28194477

[10] Chueh JY, Wakhloo AK, Hendricks GH, Silva CF, Weaver JP, Gounis MJ. Mechanical Characterization of Thromboemboli in Acute Ischemic Stroke and Laboratory Embolus Analogs. AJNR Am J Neuroradiol. 2011;32(7):1237–1244. doi: 10.3174/ajnr.A2485 21596804 PMC7966072

[11] Merritt W, Holter AM, Beahm S, et al. Quantifying the mechanical and histological properties of thrombus analog made from human blood for the creation of synthetic thrombus for thrombectomy device testing. Journal of NeuroInterventional Surgery. 2018;10(12):1168–1173. doi: 10.1136/neurintsurg-2017-013675 29695602 PMC9188866

[12] Gebrezgiabhier D, Liu Y, Reddy AS, et al. A human brain test bed for research in large vessel occlusion stroke. Journal of Neurosurgery. 2021;1(aop):1–9. doi: 10.3171/2020.7.JNS202278 33482637

[13] Staessens S, Denorme F, Francois O, et al. Structural analysis of ischemic stroke thrombi: histological indications for therapy resistance. Haematologica. 2020;105(2):498–507. doi: 10.3324/haematol.2019.219881 31048352 PMC7012484

[14] Mengozzi L, Widimský P. The potential value of histological analysis of thrombi extracted through mechanical thrombectomy during acute ischemic stroke treatment. Anatol J Cardiol. 2020;23(5):254–259. doi: 10.14744/AnatolJCardiol.2020.81342 32352416 PMC7219304

[15] Maekawa K, Shibata M, Nakajima H, et al. Erythrocyte-Rich Thrombus Is Associated with Reduced Number of Maneuvers and Procedure Time in Patients with Acute Ischemic Stroke Undergoing Mechanical Thrombectomy. Cerebrovasc Dis Extra. 2018;8(1):39–49. doi: 10.1159/000486042 29402828 PMC5836222

[16] Fitzgerald S, Mereuta OM, Doyle KM, et al. Correlation of imaging and histopathology of thrombi in acute ischemic stroke with etiology and outcome. Journal of neurointerventional surgery. 2017;9(6):529. doi: 10.1136/neurintsurg-2016-012391 27166383 PMC6697418

[17] Gersh KC, Nagaswami C, Weisel JW. Fibrin network structure and clot mechanical properties are altered by incorporation of erythrocytes. Thrombosis and haemostasis. 2009;102(6):1169. doi: 10.1160/TH09-03-0199 19967148 PMC2840711

[18] Weafer FM, Duffy S, Machado I, et al. Characterization of strut indentation during mechanical thrombectomy in acute ischemic stroke clot analogs. J NeuroIntervent Surg. 2019;11(9):891–897. doi: 10.1136/neurintsurg-2018-014601 30661030

[19] Niesten JM, van der Schaaf IC, van Dam L, et al. Histopathologic Composition of Cerebral Thrombi of Acute Stroke Patients Is Correlated with Stroke Subtype and Thrombus Attenuation. PLoS One. 2014;9(2). doi: 10.1371/journal.pone.0088882 24523944 PMC3921255

[20] Ds Liebeskind, Sanossian N, Yong Wh, et al. CT and MRI early vessel signs reflect clot composition in acute stroke. Stroke. 2011; 42 (5) doi: 10.1161/STROKEAHA.110.605576 21393591 PMC3094751

[21] Jc Benson, St Fitzgerald, Kardivel R, Johnson C, Dai D, Karen D, et al. Clot permeability and histopathology: is a clot’s perviousness on CT imaging correlated with its histologic composition? J NeuroIntervent Surg. 2020; 12(1). doi: 10.1136/neurintsurg-2019-014979 31239329 PMC7744246

[22] Gunning GM, McArdle K, Mirza M, Duffy S, Gilvarry M, Brouwer PA. Clot friction variation with fibrin content; implications for resistance to thrombectomy. Journal of neurointerventional surgery. 2018;10(1). doi: 10.1136/neurintsurg-2016-012721 28044009

[23] Moftakhar P, English JD, Cooke DI, Kim WT, Stout C, Smith WS, et al. Density of thrombus on admission CT predicts revascularization efficacy in large vessel occlusion acute ischemic stroke. Stroke. 2013, 44(1). doi: 10.1161/STROKEAHA.112.674127 23111438

[24] Soize S, Pierot L, Mirza M, et al. Fast Stent Retrieval Improves Recanalization Rates of Thrombectomy: Experimental Study on Different Thrombi. AJNR Am J Neuroradiol. 2020;41(6):1049–1053. doi: 10.3174/ajnr.A6559 32409312 PMC7342735

[25] Brouwer PA, Brinjikji W, de Meyer SF. Clot Pathophysiology: Why Is It Clinically Important? Neuroimaging clinics of North America. 2018, 28 (4). doi: 10.1016/j.nic.2018.06.005 30322597 PMC6910231

[26] Dobrocky T, Piechowiak E, Cianfoni A, Zibold F, et al. Thrombectomy of calcified emboli in stroke. Does histology of thrombi influence the effectiveness of thrombectomy? Journal of neurointerventional surgery. 2018;10(4). doi: 10.1136/neurintsurg-2017-013226 28798266

