# Supplementary material for: Novel synthetic clot analogs for in-vitro stroke modelling
Source: PLoS One. 2022 Sep 9;17(9):e0274211. doi: 10.1371/journal.pone.0274211 (PMC9462564; doi:10.1371/journal.pone.0274211)
Supplement: S1 Table — Clots that failed to interact with the clot removal device (Score = 0 for the category device integration) were classified as not suitable for an in-vitro experimental setting and thus excluded (marked in grey, n = 9). (DOCX) [file pone.0274211.s001.docx]

**S1 Table. Qualitative scores obtained for each of the 23 evaluated clots**. Clots that failed to interact with the clot removal device (Score=0 for the category *device integration)* were classified as not suitable for an in-vitro experimental setting and thus excluded (marked in grey, n=9).
