# Supplementary material for: Novel synthetic clot analogs for in-vitro stroke modelling
Source: PLoS One. 2022 Sep 9;17(9):e0274211. doi: 10.1371/journal.pone.0274211 (PMC9462564; doi:10.1371/journal.pone.0274211)
Supplement: S1 Fig — (a-d). Silicone-based clots mixed with 30% MCI/MI with (c,d) and without micro-glass beads (a,b): before (a,c) and after (b,d) mechanical sheer stress showing different breakability and elasticity degrees. (DOCX) [file pone.0274211.s002.docx]

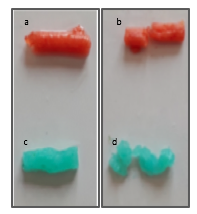


**S1 Fig. (a-d).** **Silicone-based clots mixed with 30% MCI/MI with (c,d) and without micro-glass beads (a,b):** before (a,c) and after (b,d) mechanical sheer stress showing different breakability and elasticity degrees.
