# Supplementary material for: Novel synthetic clot analogs for in-vitro stroke modelling
Source: PLoS One. 2022 Sep 9;17(9):e0274211. doi: 10.1371/journal.pone.0274211 (PMC9462564; doi:10.1371/journal.pone.0274211)
Supplement: S2 Fig — (a-c). Retrieved clots with stent retriever. Agarose-based clots mixed with 10% MCI/MI (a, c), with spiral (a) or barbed (c) supporting structures, and with 20% MCI/MI (b) were successfully retrieved with a clot extraction device (SolitaireTM 4mm x 20mm, Medtronic, Dublin, Ireland). (DOCX) [file pone.0274211.s003.docx]

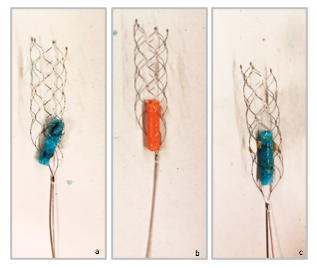


**S2 Fig (a-c).** **Retrieved clots with stent retriever.** Agarose-based clots mixed with 10% MCI/MI (a,c), with spiral (a) or barbed (c) supporting structures, and with 20% MCI/MI (b) were successfully retrieved with a clot extraction device (Solitaire^TM^ 4mm x 20mm, Medtronic, Dublin, Ireland).
